# Effect of emergency medical service response time on fatality risk of freeway crashes: Bayesian random parameters spatial logistic approach

**DOI:** 10.3389/fpubh.2024.1453788

**Published:** 2024-10-23

**Authors:** Peng Huang, Sheng Ouyang, Han Yan, Xiaofei Wang, Jaeyoung Jay Lee, Qiang Zeng

**Affiliations:** ^1^Guangzhou Expressway Co., Ltd., Guangzhou, China; ^2^School of Civil Engineering and Transportation, South China University of Technology, Guangzhou, China; ^3^School of Traffic and Transportation Engineering, Central South University, Changsha, China

**Keywords:** emergency medical service, response time, fatality risk, freeway crash, random parameters spatial logistic model

## Abstract

**Introduction:**

Emergency medical service (EMS) serves as a pivotal role in linking injured road users to hospitals via offering first aid measures and transportation. This paper aims to investigate the effect of emergency medical service (EMS) response time on the fatality risk of freeway crashes.

**Methods:**

Crash injury severity data from Kaiyang Freeway, China in 2014 and 2015 are employed for the empirical investigation. A Bayesian random parameters spatial logistic model is developed for analyzing crash severity.

**Results:**

Bayesian inference of the random parameters spatial logistic model demonstrates the importance of reducing EMS response time on minimizing the fatality risk of freeway crashes. Fatality odds would increase by 2.6% for 1 min increase in EMS response time. Additionally, vehicle type, crash type, time of day, horizontal curvature, vertical grade, and precipitation are also found to have significant effects on the fatality probability of freeway crashes.

**Conclusion:**

It is crucial to reduce EMS response time to decrease the fatality likelihood of freeway crashes. Some countermeasures have been proposed to shorten EMS response time.

## 1 Introduction

Given the huge emotional and economic burden imposed by roadway crashes on the society, identifying contributing factors to crash occurrence and injury severity and quantifying their effects have long been a research focus in the research field of traffic safety (1). Most of existing studies (2–5) explored contributing factors to crash occurrence (pre-crash factors) and those to injury severity during the crash event from human, vehicle, and environmental factors. On the other hand, post-crash factors, such as those pertaining to emergency medical services (EMS), have been relatively less investigated, due to data availability and reliability.

After the crash occurrence, EMS serves as a pivotal role in linking injured road users to hospitals via offering first aid measures and transportation (6). EMS response time, herein defined as the time that elapses from the notification of a traffic crash until EMS personnel arrives at the crash scene (7), is an important indicator of the time for patients to be treated and the performance of dispatching EMS resources (8). Many safety researchers (9–11) acknowledge the significant impact of EMS response time on the fatality likelihood of traffic crashes, especially in cases of victims sustaining severe injury. This opinion is reasonable, because a proportion of crash deaths would be evitable, from the clinical perspective, if timely medical treatments were provided to the severely injured victims, particularly those with brain/heart trauma (12) or in need of haemorrhage controls or open airways (13, 14).

Despite of sparseness, there is a small quantity of studies that have investigated the effect of EMS response time on the injury severity of traffic crashes, using discrete outcome models. For example, based on a binary probit regression analysis of a dataset with over 1,400 traffic crashes in Spain, Sánchez-Mangas et al. (11) concluded that a 10-min reduction of EMS response time is expected to result in the fatality likelihood decreasing by 33%. Using a dataset from the Fatality Analysis Reporting System (FARS) in the U.S., Ma et al. (15) investigated the smooth relationship between crash fatality probability and EMS response time and found that 17 min is the gold time for crash rescues. Lee et al. (6) developed a random effects ordered probit model for analyzing crash injury severity, and incorporated crash-reporting time, response time, and transport time into the analysis. Their results indicate that longer response time and transport time are linked to more severe injury outcomes. Hosseinzadeh and Kluger (16) also adopted a random effects ordered probit model for uncovering the association between crash injury severity and EMS response time and on-scene time, as well as crash-related factors. They found that shorter response time and longer on-scene time is helpful to decrease the severity level of entire-body injuries. Zeng et al. (17) proposed a spatial generalized ordered probit model for the analysis of freeway crash severity, where EMS response time is included as an explanatory variable. The results also suggested that is lower crash severity is associated with a shorter EMS response time. Although the above studies have all demonstrated the significant impact of EMS response time on crash injury severity, a distinct difference lies in them: the former two studies categorized crash severity into two levels (i.e., fatal and non-fatal) and assumed that EMS response time has a considerable effect on fatality likelihood, and the latter three studies categorized crash severity into more than two levels (e.g., KABCO has five severity levels) and implied that EMS response time may have significant effects on the likelihoods of other severity levels, such as no injury. Obviously, the former assumption is more reasonable and consistent to the findings from clinic medicine. To accurately quantify the effect of EMS response time on the fatality likelihood of freeway crashes, in this research, the injury severity will be divided into fatal and non-fatal, as in Sánchez-Mangas et al. (11) and Ma et al. (15). Our research focuses on freeway crashes, as they are more likely to result in human deaths than traffic crashes on other types of roadways, such as urban roads. Besides, the unique built environment (e.g., far from hospitals) and roadway conditions (e.g., the existence of emergency lane and no intersection) may make the EMS response time for freeway crashes different from that for crashes on other roadways.

Analytic method is also important to analyze crash injury severity. Given the binary categorization, statistically, binary logit or probit models have been most frequently used. In the recent decade, accounting for the unobserved heterogeneity and spatial correlation is prevalent when modeling crash severity (18, 19). Random parameters (20), latent class/finite mixture (21), and Markov switching approaches (22) are typical methods capable of capturing the unobserved heterogeneity. Among them, random parameters approaches are most widely used. To accommodate spatial correlation, various spatial structures, including spatial lag (23, 24), spatial error (23), intrinsic conditional autoregressive (CAR) (25, 26), and Leroux CAR (17), have been incorporated into the formulation of discrete outcome models. Zeng et al. (17) found that the Leroux CAR is superior to other alternatives. In this research, we propose a random parameters spatial logistic model with Leroux CAR for analyzing freeway crash severity, which can simultaneously capture the unobserved heterogeneity and spatial correlation in it.

The rest of the article is organized into four sections. The freeway crash-severity data used for the empirical analysis are introduced in Section 2. We specify the formulation of the random parameters spatial logistic model in Section 3. The Bayesian estimation results of the proposed model are summarized and interpreted in Section 4. In the last section, we draw conclusions from the research and offer guidance for future research.

## 2 Data

Crash data of 2 years in 2014 and 2015 were collected from Kaiyang Freeway in Guangdong, China, which were acquired from the Highway Maintenance and Administration Management System maintained by Guangdong Transportation Group. Excluding the crash records with incomplete information, 1,414 crash records were used for the empirical analysis. In the original crash records, injury severity is categorized into four levels: no injury, slight injury, severe injury, and fatality. As mentioned earlier, the paper focuses on quantifying the effect of EMS response time on the fatality likelihood of freeway crashes versus that of non-fatality. Thus, the injury severity in the analysis was contracted into two levels: non-fatality (combining no injury, slight injury, and severe injury) and fatality. Among the observations, 1,378 crashes’ severity levels are of non-fatality and 36 crashes’ are of fatality.

**Figure 1:**
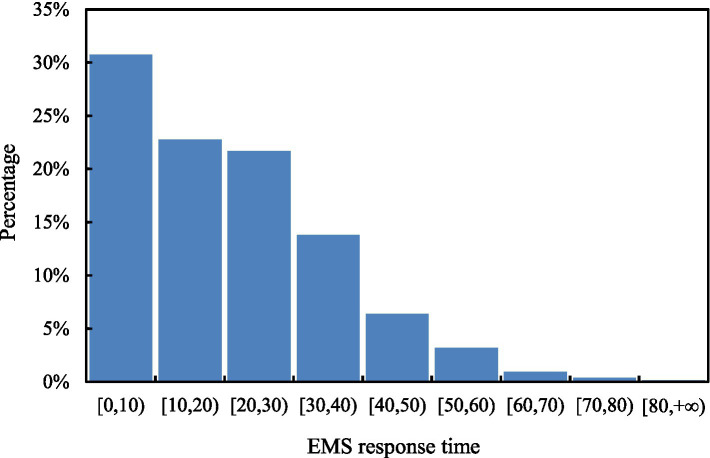
The distribution of EMS response time.

In the crash records, in addition to injury severity, some information pertaining to EMS, involved vehicle(s) and accident configuration is also documented, including: EMS response time, vehicle type (passenger car, coach, truck, and others) and license number (which is used to distinguish if a vehicle is local or not), crash time (morning, afternoon, evening, and before dawn), crash date (weekday and weekend), crash type (single-vehicle crash, rear-end crash, and angle crash), and crash location (which is expressed as kilometers marker of the freeway). As the key factor under investigation, the distribution of EMS response time in the dataset is shown in Figure 1.

We obtain the geometry design materials on Kaiyang Freeway from Guangdong Province Communication Planning and Design Institute Co., Ltd. Four roadway attributes, including horizontal curvature, vertical grade, and if the crash site is near a ramp or on a bridge, are extracted from the materials and matched with each crash according to their location information. To capture the spatial effects in the crashes, the freeway is segmented into 154 sections based on the criterion of homogeneity in horizontal curvature and vertical grade, which is line with the roadway segmentation methods used in the past studies (17, 27).

We collect the real-time weather data along the freeway from the Meteorological Information Management System. The system is administrated by the Guangdong Climate Centre, an official meteorological organization. The data on wind speed, precipitation, visibility, temperature, and humidity are observed and recorded in each hour by three weather stations: Enping Weather Station, Kaiping Weather Station, and Yangjiang Weather Station. We match each crash under investigation with the hourly meteorological information from the nearest weather station through the crash time and location.

Table 1 displays the definitions and descriptive statistics of crash injury severity and its potential influence factors for the empirical analysis. We conduct Pearson correlation test and multi-collinearity diagnoses for the factors in SPSS software and find that there is no significant correlation or multi-collinearity among them.

**Table 1:** Definitions and descriptive statistics of crash injury severity and influence factors.

Variables	Description	Mean	S.D.	Min.	Max.
Crash injury severity	Fatality = 1; non-fatality = 0	0.025	0.155	0	1
EMS response time	The duration from the crash notification to the arrival of EMS personnel at crash scene (minute)	19.56	16.61	1	260
Weekend	Crash happens on a weekend = 1; otherwise = 0	0.347	0.476	0	1
Vehicle characteristics					
Car^*^	Only passenger cars are involved =1; otherwise = 0	0.579	0.494	0	1
Coach	At least a coach is involved =1; otherwise = 0	0.064	0.245	0	1
Truck	At least a truck is involved = 1; otherwise = 0	0.313	0.464	0	1
Other_vehicle	At least a other vehicles (e.g., towed vehicles) is involved = 1; otherwise = 0	0. 098	0.298	0	1
Non_local_vehicle	At least an involved vehicle is not registered in Guangdong Province (non-local vehicle) = 1; otherwise (local vehicle) = 0	0.280	0.433	0	1
Time of day					
Before_dawn^*^	Crash happens within the period [00:00, 06:00) = 1; otherwise = 0	0.223	0.416	0	1
Morning	Crash happens within the period [06:00, 12:00) = 1; otherwise = 0	0.370	0.483	0	1
Afternoon	Crash happens within the period [12:00, 18:00) = 1; otherwise = 0	0.223	0.418	0	1
Evening	Crash happens within the period [18:00, 24:00) = 1; otherwise = 0	0.184	0.388	0	1
Crash type					
Single-vehicle crash^*^	Only one vehicle is involved in the crash = 1; otherwise = 0	0.455	0.498	0	1
Rear-end crash	A rear end collision = 1; otherwise = 0	0.381	0.486	0	1
Angle crash	An angle collision = 1; otherwise = 0	0.163	0.245	0	1
Roadway attributes					
Curvature	The horizontal curvature of crash location (0.1 km-1)	1.838	1.233	0	4.35
Grade	The vertical grade of crash location (%)	0.709	0.588	0	2.91
Bridge	Crash happens on a bridge =1; otherwise = 0	0.536	0.499	0	1
Ramp	Crash happens near a ramp = 1; otherwise = 0	0.244	0.430	0	1
Dynamic weather conditions					
Wind speed	Average wind speed during the hour of crash occurrence (m/s)	2.860	1.889	0	16.7
Precipitation	Accumulated precipitation during the hour of crash occurrence (mm)	0.760	3.425	0	54.8
Temperature	Average air temperature during the hour of crash occurrence (°C)	23.68	6.057	4.8	36.8
Humidity	Average humidity during the hour of crash occurrence (%)	81.31	15.48	21	100
Visibility	Average visibility during the hour of crash occurrence (km)	17.77	18.41	0.1	80

* The reference category.

## 3 Methods

We propose a random parameters spatial logistic model for investigating the crash injury severity with binary outcomes (fatality and non-fatality). To justify the superiority of the proposed model, we compare it with traditional logistic model and spatial logistic model. In the section, the formulations of these models are clearly specified in the order of model complexity (Section 3.1); and then the implementation processes of Bayesian estimation and performance assessment criterion for the models are introduced (Section 3.2).

### 3.1 Model formulation

#### 3.1.1 Logistic model

Logistic model (i.e., binary logit model) is one of the most extensively used methods for the analysis of crash injury severity divided into two levels (1). As suggested in Table 1, denote fatal crash = 1 and non-fatal crash = 0. For any crash i , a latent variable $U_{i}$ is set. It is assumed that there is a linear association between $U_{i}$ and the covariates. If $U_{i}$ is positive, the injury severity of crash i is fatal; otherwise, it is non-fatal. The model equation is shown in Equations 1 and 2:


(1) $$U_{i}=\beta_{0}+\overset{J}{\underset{j=1}{\sum }}\beta_{j}x_{i,j}+\varepsilon_{i}\text{,}$$

(2) $$Y_{i}=\{\begin{array}{cc} 1, & \mathrm{if}\;U_{i}>0\\ 0, & \mathrm{if}\;U_{i}\leq 0 \end{array}\text{,}i=1,2,\cdots ,N$$

where $x_{i,j}$ is the observed value of the j th $\left(j=1,2,\cdots ,J\right)$ covariate, $x_{j}$ , in crash i , and $\beta_{j}$ is the coefficient corresponding to $x_{j}$ . $\beta_{0}$ is a constant term. J and N are the numbers of covariates and crashes in the dataset. $\varepsilon_{i}$ is a residual term and is assumed to have a logistic distribution. Its cumulative distribution function is expressed as Equation 3:


(3) $$F\left(\varepsilon \right)=\frac{1}{1+\exp\left(-\varepsilon \right)}\text{,}$$

According to the model formulation, the probabilities of crash i resulting in fatality and non-fatality (represented by $p_{i,1}$ and $p_{i,0}$ respectively), can be calculated as Equations 4 and 5:


(4) $$p_{i,1}=\frac{\exp\left(\beta_{0}+\sum_{j=1}^{J}\beta_{j}x_{i,j}\right)}{1+\exp\left(\beta_{0}+\sum_{j=1}^{J}\beta_{j}x_{i,j}\right)}\text{,}$$

(5) $$p_{i,0}=\frac{1}{1+\exp\left(\beta_{0}+\sum_{j=1}^{J}\beta_{j}x_{i,j}\right)}\text{,}$$

Accordingly, the odds of fatality crash are calculated as Equation 6:


(6) $$p_{i,1}/p_{i,0}=\exp\left(\beta_{0}+\overset{J}{\underset{j=1}{\sum }}\beta_{j}x_{i,j}\right)\text{,}$$

To quantify the effect of a certain factor on crash injury severity, its odds ratio is usually computed and reported (28). For any covariate $x_{j}$ , its odds ratio is defined as Equation 7:


(7) $$OR_{j}=\frac{\left(p_{i,1}/p_{i,0}|x_{i,1},\cdots ,x_{i,j}+1,\cdots ,x_{i,J}\right)}{\left(p_{i,1}/p_{i,0}|x_{i,1},\cdots ,x_{i,j},\cdots ,x_{i,J}\right)}=\exp\left(\beta_{j}\right)\text{.}$$

#### 3.1.2 Spatial logistic model

Some unobserved/unobservable factors may have similar effects on the injury severities of crashes in close proximity, resulting in spatial correlation/dependency across them (17). To account for the spatial dependency, a spatial logistic model is developed, by adding a random error term with CAR prior into the formulation of $U_{i}$ . Different from the intrinsic CAR prior adopted in the previous studies (25, 26), the Leroux CAR prior which is able to flexibly capture the strength of spatial correlation (29), is specified in the spatial logistic model. Specifically, for crash i occurring in freeway section m can be calculated by Equations 8 and 9:


(8) $$U_{i}=\beta_{0}+\overset{J}{\underset{j=1}{\sum }}\beta_{j}x_{i,j}+\varepsilon_{i}+\varphi_{m}\text{,}$$

(9) $$\varphi_{m}\sim N\left(\frac{\rho \sum_{n\neq m}\omega_{m,n}\varphi_{n}}{1-\rho +\rho \sum_{n\neq m}\omega_{m,n}},\frac{\delta^{2}}{1-\rho +\rho \sum_{n\neq m}\omega_{m,n}}\right)\text{,}m=1,2,\cdots ,M$$

where $\varphi_{m}$ and $\varphi_{n}$ represent the spatial effects of crashes in freeway sections m and n . $\omega_{m,n}$ represents the degree of the proximity between sections m and n . The first order adjacency-based rule which is widely used in spatial modeling, is employed to define the proximity degrees: if freeway sections m and n share a common end, $\omega_{m,n}=1$ ; otherwise, $\omega_{m,n}=0$ . $\rho \left(0\leq \rho \leq 1\right)$ is an estimable parameter which measures the strength of spatial correlation. A higher value of ρ indicates stronger spatial correlation. ρ=0 implies that no spatial correlation exists among the injury severities of observed crashes. ρ=1 (equivalent to the intrinsic CAR prior) suggests that the injury severities of adjacent crashes are fully correlated. δ is a hyper-parameter related to the variance of spatial correlation.

#### 3.1.3 Random parameters spatial logistic model

There may be unobserved heterogeneities in the effects of certain factors on crash injury severity (18). To simultaneously account for unobserved heterogeneity and spatial correlation, a random parameters spatial logistic model is proposed. Specifically, the coefficient $\beta_{j}\left(j=0,1,2,\cdots ,J\right)$ in Equation 8 is switched to random parameters $\beta_{i,j}\left(i=1,\;2,\cdots ,\;N;\;j=0,\;1,\;2,\cdots ,\;J\right)$ which can vary across crashes. Although there are a number of applicable forms of random parameters, the commonest one is adopted in the study as shown in Equation 10:


(10) $$\beta_{i,j}={\bar{\beta }}_{j}+\mu_{i,j}\text{,}$$

in which ${\bar{\beta }}_{j}$ is the mean of $\beta_{i,j}$ . $\mu_{i,j}$ is a normally distributed random term as shown in Equation 11:


(11) $$\mu_{i,j}\sim N\left(0,\sigma_{j}^{2}\right)\text{,}$$

where $\sigma_{j}$ denotes the standard deviation of $\mu_{i,j}$ .

### 3.2 Model estimation and performance assessment criterion

#### 3.2.1 Model estimation

Given the complex structure of CAR prior, the above models are estimated by Bayesian inference method. It necessitates specifying a prior distribution for each parameter or hyper-parameter, which reveals the researchers’ prior knowledge on it. In the case of no available prior knowledge, as in the previous studies (17, 25, 26), non-informative distributions are adopted. Specifically, a diffused normal distribution, $N\left(0,10^{4}\right)$ , is set as the prior of $\beta_{j}$ and ${\bar{\beta }}_{j}\left(j=0,1,2,\cdots ,J\right)$ ; a uniform distribution, $U\left(0,1\right)$ , is set as the prior of ρ ; and a uniform distribution, $U\left(0.01,10\right)$ , is set as the prior of δ and $\sigma_{j}\left(j=0,1,2,\cdots ,J\right)$ .

**Figure 2:**
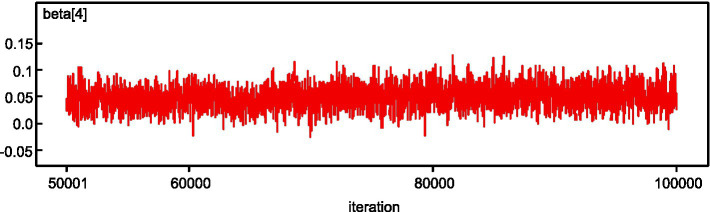
History plot of the parameter for EMS response time in the random parameters model.

The Bayesian estimation is implemented in the WinBUGS software (30), in which Gibbs sampling algorithms and Markov chain Monte Carlo (MCMC) simulation techniques are embedded to infer the posterior distributions of parameters. For each model, a chain of MCMC simulation is run, and 100,000 simulation iterations are set, with the first 50,000 iterations acting as burn-in. To judge if the MCMC simulations are converged, we visually inspect the history plots for the parameters (such as that for EMS response time in the random parameters model as shown in Figure 2) and monitor whether the ratio between the Monte Carlo simulation error for each parameter and its posterior standard deviation is less than 5%. In the random parameters spatial logistic model, if the posterior variance is not statistically significant at the 95% Bayesian credibility level, it is transformed to a fixed parameter.

#### 3.2.2 Performance assessment criterion

The deviance information criterion (DIC), which is the most popular criterion for assessing Bayesian models, is used for comparing the comprehensive performance of the above crash severity models. As a Bayesian generalization of Akaike’s information criterion, the DIC provides a hybrid measure of model fitting and complexity. According to Spiegelhalter et al. (31), its calculation equation is expressed as Equation 12:


(12) $$DIC=\bar{D}+pD\text{,}$$

where $\bar{D}$ and pD are the posterior mean deviance and the effective number of parameters respectively, which are used to measure the model fitting and complexity accordingly. Generally, a lower DIC value means a better overall performance. As suggested by Lunn et al. (30), we can conclude that a model with a lower DIC is considerably superior if the DIC difference with another one is greater than 10.

## 4 Modeling results

The results of Bayesian estimation and performance assessment for the three models are summarized in Table 2. Only the covariates whose parameters are significant at least at the 90% credibility level are included in the table. We can find that the $\bar{D}$ value of the spatial logistic model is lower than that of the logistic model with the difference over 20. It indicates that the spatial logistic model performs substantially better than the logistic model in fitting the association between crash severity and EMS response time as well as other factors. Although the lower pD value of the logistic model implies that it is more parsimonious, the 16 points of DIC lower for the spatial logistic model suggest its superior overall performance. These findings are in line with the previous studies (17, 23, 25, 26): accounting for spatial correlation among adjacent crashes by CAR prior can effectively reduce model misspecification and improve model estimation. The reasonableness of the spatial logistic model with Leroux CAR prior can also be demonstrated by the Bayesian estimates of δ and ρ , which are both significant at the 95% credibility level. Additionally, the posterior mean of ρ is 0.62. It implies that the spatial correlation in the crash severity is medium, that cannot be figured out by the intrinsic CAR prior applied in Xu et al. (26) and Meng et al. (25).

**Table 2:** Results of Bayesian parameter estimation and performance assessment for the models.^a^

	Logistic model	Spatial logistic model	Random parameters spatial logistic model
Constant	−5.99 (1.87)^b^,^**^	−6.67 (1.98)^**^	−7.10 (2.09)^**^
EMS response time	0.021(0.009)^**^	0.024 (0.010)^**^	0.026 (0.010)^**^
Truck	0.41 (0.12)^**^	0.44 (0.13)^**^	0.51 (0.15)^**^
S.D. of Truck	—	—	1.23 (0.16)^**^
Other vehicle	0.64 (0.20)^**^	0.68 (0.23)^**^	0.71 (0.22)^**^
Non_local vehicle	0.87 (0.31)^**^	0.99 (0.34)^**^	0.86 (0.32)^**^
Curvature	−0.17 (0.06)^**^	−0.14 (0.05)^**^	−0.12 (0.04)^**^
Grade	0.79 (0.24)^**^	0.91 (0.29)^**^	1.09 (0.31)^**^
Afternoon	−2.53 (0.97)^**^	−2.57 (0.98)^**^	−2.67 (1.01)^**^
Rear-end crash	1.44 (0.56)^**^	1.53 (0.56)^**^	1.47 (0.54)^**^
Angle crash	1.76 (0.62)^**^	1.85 (0.62)^**^	1.91 (0.67)^**^
Precipitation	—	0.92 (0.62)^*^	0.95 (0.65)^*^
ρ	—	0.62 (0.27)^**^	0.68 (0.26)^**^
δ	—	0.74 (0.17)^**^	0.56 (0.22)^**^
$\bar{D}$	285	259	236
pD	18	28	35
DIC	303	287	271

a Weekend, Coach, Morning, Evening, Bridge, and Ramp, are excluded, because their effects on crash injury severity are not significant at the 90% credibility level in the models.

b Bayesian posterior mean of the parameter (Bayesian posterior standard deviation of the parameter).

* Statistically significant at the 90% credible level.

** Statistically significant at the 95% credible level.

The random parameters spatial logistic model yields the lowest values of $\bar{D}$ and DIC. We may conclude that accounting for the unobserved heterogeneities in the effects of certain covariates by allowing their parameters to vary across observations can further improve model fitting performance, given the consideration of spatial correlation. Similar results can be found in the research conducted by Zeng et al. (32). In the random parameters spatial logistic model, the posterior mean of ρ is a little higher than the counterpart in the spatial logistic model. That is, the strength of spatial correlation is slightly increased due to the accommodation of random parameters. Besides, the posterior mean of δ is significantly lower in the random parameters model. This is reasonable, as a proportion of the structure spatial effects may be derived from the unobserved heterogeneity which has been captured by the random parameter.

## 5 Discussion

The effects of EMS response time and other significant variables on the fatality probability of freeway crashes are interpreted based on the parameter estimation in the random parameters spatial logistic model, since it outperforms the other two models.

### 5.1 Effect of EMS response time

According to the Bayesian modeling estimation results summarized in Table 2, the parameter for *EMS response time* is statistically significant with a positive coefficient at the 95% credibility level, which indicates that a longer *EMS response time* is expected to increase the probability of fatality crash. The odds ratio for *EMS response time* is estimated to be 1.026 (=exp.(0.026)), i.e., the odds of resulting in fatality crash would be increased by 2.6% for per one-minute increase of *EMS response time*. The findings are generally consistent with those in most of the previous studies (6, 11, 17) and experiences from clinic medicine and transportation engineering: rapid response of EMS personnel is able to prevent the death of certain traffic crash victims suffered from severe trauma injuries, but are different from those in some others. For example, Ma et al. (15) found that the effect of EMS response time on fatality risk is non-monotonic, such that EMS response time may be negatively associated with the odds of fatality in some cases. These phenomena are originated from the urgency level of a crash and EMS dispatch priority (15, 33–35). Nevertheless, we did not find such phenomena from the current study. It is possible because freeway crashes in China usually have the high priority for EMS dispatch, given their more severe outcomes than those on other types of roadways (17). Besides, the average marginal effect of 10-min EMS response time on fatality crash is estimated to be 0.0023^1^. That is, 0.23 less fatality crash per every 100 crashes is expected for a 10-min reduction in EMS response time. The marginal effect is significantly lower than that (=0.024) estimated by Sánchez-Mangas et al. (11), which may be attributed to lower fatality rate of our crash data and the differences in EMS level between China and Spain.

**Figure 3:**
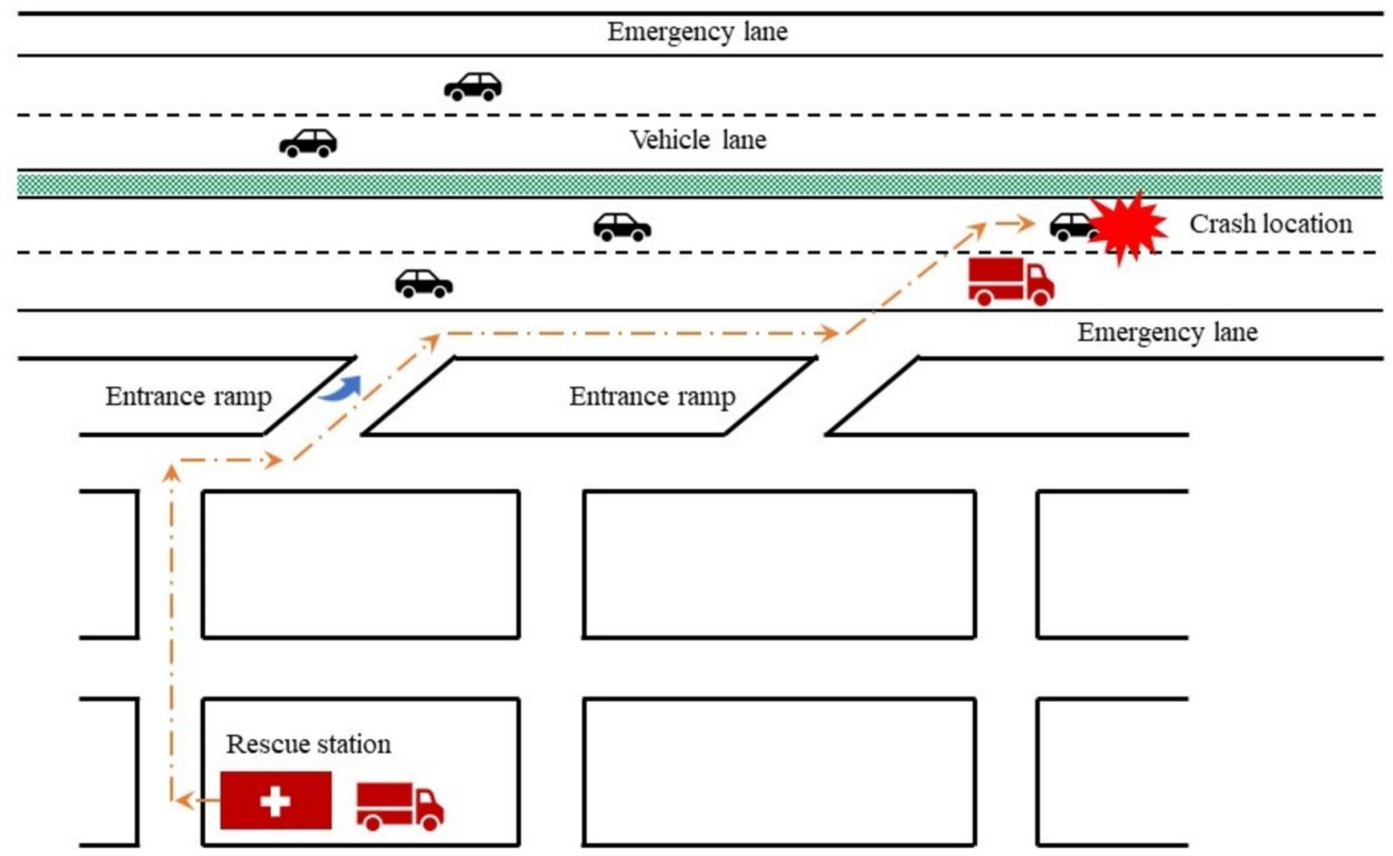
Traveling path of a rescue vehicle.

To make the EMS personnel and vehicles arrive at crash scenes as soon as possible, the following countermeasures may be effective (1): Installing sufficient EMS facilities near freeways. Once a freeway crash is reported or detected, the emergency management agencies usually dispatch rescue personnel and vehicles from the nearest EMS facility to the crash scene. More EMS facilities can reduce the expected distance between them and crash locations (2).; Optimizing the traveling path to crash scenes (as shown in Figure 3), according to the real-time traffic data collected by various detection techniques and transmitted by 5G communication technology. There may be several alternative traveling paths from a EMS facility to the crash scene. The travel time of each path is depended on the traffic conditions which is usually influenced by the traffic crash. If the traffic conditions on each path is detected, transmitted, and predicted in real-time, we can find the one with shortest travel time (3). Strict enforcement against illegal occupancy of emergency lanes on freeways. The occurrence of traffic crash may bring about traffic congestion. A rescue vehicle can travel on emergency lanes to avoid the adverse impacts of traffic congestion on its travel speed. However, emergency lanes may also be illegally occupied by other vehicles. Thus, strict enforcement against the illegal occupancy is also helpful to reduce the arrival time of EMS vehicles.

### 5.2 Effects of other factors

Truck is the only covariate that has a heterogeneous effect on crash injury severity. The estimated mean and standard deviation of the random parameter for *truck* are 0.51 and 1.23 respectively, which indicate that about 66% of truck involved crashes are more likely to result in fatality. It is reasonable, because trucks usually possess larger mass and higher structural rigidity which impose greater harm on the occupants in other vehicles involved in the same collision, i.e., higher crash aggressivity defined in Huang et al. (36). Meanwhile, the rest 34% of truck involved crashes are less likely to result in fatality. In this research, a proportion of *truck’s* effect on crash severity may be derived from truck drivers’ driving behavior which is not observed in the crash data. Considerable variability in truck drivers’ behavior when occurring a crash may explain the heterogeneous effect.

*Other_vehicle* is found to have a significant and homogeneous effect on crash severity. According to the Bayesian estimates, the fatality odds of crashes involving other type vehicles (e.g., towed vehicles) are 2.03 [=exp(0.71)] times of that of crashes involving passenger cars only, with all other factors equal. Similar to trucks, other type vehicles also possess higher crash aggressivity than passenger cars, thus more likely to resulting in fatalities.

It is interesting to find that *non_local* vehicle has a significantly positive effect on crash severity. That is, involving non-local vehicles (i.e., those not registered in the province where the crash happened) would increase the crash fatality risk. Specifically, the fatality odds are expected to increase by 136% [=exp(0.86)−1], if there is one or more non-local vehicle involved in a crash. We can find similar results in the research conducted by Zeng et al. (17). They argued that the drivers of non-local vehicles are usually less familiar with the roadway environment, and thus may not have enough time to take proper actions before crashes.

Regarding roadway geometric attributes, *curvature* and *grade* are significantly associated with crash injury severity. The negative sign of the parameter for *curvature* suggests that crashes on freeway sections with smaller horizontal curve radius are less likely to result in fatalities. The crash fatality odds would decrease by 11% [=1−exp(−0.12)] for a 0.1 km^−1^ increase in horizontal curvature. It is possible, because drivers tend to reduce speed and become more cautious to avoid vehicles out of control when driving on small radius curves (37, 38). The estimated mean of the parameter for *grade* is 1.09. It indicates that the odds of crash fatality would increase by 197% [=exp(1.09)−1] for a 1% increase in vertical grade. High grade would reduce sight distance (2, 5). Thereby, less time is retained for drivers to appropriately respond to upcoming crashes.

For the time of day, the parameter for *afternoon* on crash injury severity is negative at the 95% credibility level. It is anticipated, as the vision of drivers is usually clearer in afternoon than before dawn (the reference case), and thus more time is available for them to take defensive actions when encountered with emergency. In addition, because of the light traffic and human circadian rhythmicity respectively, we may observe more frequent speeding and fatigue/drowsy driving before dawn, which probably result in severe traffic accidents (4).

With regard to crash type, the estimation results suggest that rear-end crashes and angle crashes are more prone to lead to fatalities than single-vehicle crashes (the reference type). Particularly, the fatality odds of rear-end crashes and angle crashes are 4.35 and 6.75 times of that of single-vehicle crashes, respectively. The results are generally in line with the findings of Zeng et al. (17), and may be attributed to that more casualties usually exist in multiple-vehicle crashes (covering rear-end crashes, angle crashes, and others) than in single-vehicle crashes.

*Precipitation* is the weather-related variable with a significant effect on crash injury severity. According to its estimated parameter, heavier precipitation is associated with higher probability of fatality crash. The results are consistent with the previous research (39, 40) and engineering intuitions: because of rainfall, roadway surfaces would become slippery and their skidding resistance would be reduced. Accordingly, vehicles would collide at higher speeds which were prone to bring about severer injury severity outcomes. Additionally, during the processes of precipitation, drivers’ vision might be impaired which results in reduced reaction time available to drivers.

## 6 Conclusion

This research empirically investigated the effect of EMS response time and the fatality risk of freeway crashes, using a two-years crash injury severity dataset from Kaiyang Freeway, China. A Bayesian random parameters spatial logistic model was advocated for the empirical investigation. The advocated model simultaneously accounted for the spatial correlation across adjacent crashes and unobserved heterogeneities in effects of the observed factors.

The values of DIC indicated that the overall performance of the random parameters spatial logistic model is substantially better than the logistic model and spatial logistic model. The parameter estimation results in the random parameters spatial model revealed that EMS response time has a significantly positive effect on crash injury severity. One minute increase in EMS response time would increase the crash fatality odds by 2.6%. Three countermeasures were suggested to reduce the EMS response time. They are: (1) establishment of EMS facilities near freeways at the optimized location; (2) optimization of path to the crash location based on real-time traffic data; and (3) strict enforcement against illegal occupancy of emergency lanes.

In addition, the estimation results show that truck has a heterogeneous effect on crash injury severity. Fatalities are more likely to occur in crashes involving other vehicles, non-local vehicles, on freeway sections with smaller horizontal curvature and greater vertical grade, in weather conditions with more precipitation, and before dawn. The fatality risk of rear-end crashes and angle crashes is higher than that of single-vehicle crashes.

While the significant effect of EMS response time on crash fatality risk and the superiority of the advocated Bayesian random parameters spatial logit model were demonstrated, there are some limitations to the current research. For instance, only the crash data from one freeway are used in the model development, and the attributes related to drivers are not included. It will be necessary to further validate the safety effect of EMS response time if comprehensive crash injury severity data are available in the future.

## Data Availability

The data are available from the corresponding authors upon reasonable request. Requests to access these datasets should be directed to Qiang Zeng, zengqiang@scut.edu.cn.

## Footnotes

^1^Please refer to Afghari et al. (41) for the detailed calculation method of marginal effect.
